# Design of Fault Prediction System for Electromechanical Sensor Equipment Based on Deep Learning

**DOI:** 10.1155/2022/3057167

**Published:** 2022-03-17

**Authors:** Yongtao Ding, Hua Wu, Kaixiang Zhou

**Affiliations:** Department of Mechanical Engineering, City University of Hong Kong, 999077 Hong Kong SAR, China

## Abstract

With the increasing complexity, scale, and intelligentization of modern equipment, the maintenance cost of equipment is increasing day by day. Moreover, once an unexpected major failure occurs, it will cause loss and damage to production, economy, and safety. Based on the considerations of system reliability and safety, fault prediction has gradually become a hot topic in the field of reliability. As a new branch of machine learning, deep learning realizes deep abstract feature extraction and expression of complex nonlinear relations by stacking deep neural networks and makes its methods solve bad problems in many traditional machine learning fields. The improvement and excellent results have been achieved. This article first introduces the model structure and working principle of the classic deep learning model noise reduction autoencoder and combines the feature extraction results of the experimental data of electromechanical sensor equipment and the model characteristics to analyze that this type of model can be trained using only normal samples. Under the restriction, the reason for abnormal features can also be correctly filtered. Then, in order to suppress the overfitting of training, the dropout layer is used in this article. The dropout layer will make the hidden layer nodes to be dropped with probability *p* according to the set probability. Because the lost nodes are random, each training is equivalent to training a new model. It achieves an effect similar to independently training the model and then superimposing it. Experiments prove that the dropout layer is very effective in solving overfitting. Finally, this paper conducts experimental verification on the improved algorithm. The results show that the improved model has a certain improvement in accuracy under the limited training algebra. Under the same training parameters, the accuracy is increased by approximately 2.44%, and the improved model has a better training effect and can be used for electromechanical effective prediction of sensor equipment failure.

## 1. Introduction

The structure of large-scale mechanical equipment such as high-speed EMU trains, steam turbines, and aerospace engine units is becoming more and more complex, and the probability of failure is gradually increasing. Fault diagnosis helps the system operate safely and efficiently. However, the traditional planned maintenance needs to stop production and cause high maintenance costs, and no matter how the maintenance interval is set, there is always the problem of insufficient maintenance or overmaintenance. Therefore, in consideration of factors such as reliability, safety, and economy of complex systems, equipment predictive maintenance provides maintenance engineers with a decision-making basis through early fault diagnosis and remaining life prediction so that the transition from planned maintenance to condition-based maintenance can be realized. United Airlines' predictive maintenance strategy has reduced the maintenance cost of Douglas DC-8 aircraft by 30%, and the fatality rate per 100 million passenger-kilometers has dropped from 0.6 to about 0.2. After adopting the predictive maintenance strategy, the United States' fifth-generation fighter aircraft not only reduced maintenance costs but also increased the overall reliability by more than 50%. However, the accuracy of fault diagnosis and remaining life prediction depends on the effectiveness of fault feature extraction methods. As an advanced feature extraction tool, deep learning has been widely used in natural language processing, text processing, and other fields. In recent years, it has also attracted the attention of experts and scholars in the field of fault diagnosis [1–6].

Starting from the original monitoring data of electromechanical sensor equipment, this paper studies the fault diagnosis method of electromechanical sensor equipment based on deep learning. First, build a deep neural network prediction framework based on understanding the principles of deep learning; then start from the electromechanical sensor monitoring information; study equipment fault diagnosis methods for electromechanical sensor monitoring information; and finally, on this basis, further study more complex multisensors The monitoring information fault diagnosis lays the foundation for the subsequent research on the remaining life prediction of equipment based on deep learning.

## 2. Related Work

With the development of equipment in the direction of complexity, intelligence, and integration, its working environment and operating conditions have become more and more complex, and the data collected by the monitoring system have become larger and larger, based on the current large amount of electromechanical data, the fault diagnosis of electromechanical sensor equipment has become the research focus of machine learning. It has become a hot issue in the current PHM field. Drawing on the successful experience of deep learning, some scholars have already applied deep learning in the field of fault diagnosis and have achieved certain results. Since Tamilselvan first applied DBN to aircraft engine fault diagnosis in 2013, more and more scholars at home and abroad have paid attention to this field and achieved many research results. Tran et al. merged the DBN and TKEO algorithms and applied them to the valve fault diagnosis of reciprocating compressors. The author used GRBM instead of RBM to get rid of the shortcomings of traditional RBM that can only input binary fault signals and use the DBN model as a classifier for electromechanical sensors and a traditional Residual Neural Network, a higher accuracy rate has been achieved. Jia et al. applied deep neural networks to the feature extraction and diagnosis of bearing faults. They used the feature extraction capabilities of deep neural networks to extract and identify bearing fault features directly from frequency domain signals and achieved good results. Lu et al. specially studied the fault feature extraction ability of deep neural networks in the frequency domain signals of rotating bearings and gave the visualization results of the fault features. By summarizing the above-mentioned literature, although deep learning has been successfully applied in the field of failure, the research in this field is still immature, and there are still many problems to be solved. The main problems are summarized as follows: (1) only use deep learning as a classifier, traditional signal processing methods are still used to extract fault features, and the ability of deep learning to mine fault features is not fully utilized; (2) when using deep learning algorithms to extract fault features and identify the health status of fault signals, most of the signal frequencies are used, and when the signal does not have periodicity, this method will fail and is not general; (3) the comparative study of deep learning and other shallow models is not sufficient; and (4) the role of deep learning in the fault diagnosis of electromechanical sensor monitoring information application remains to be studied [7–15].

## 3. Related Theoretical Methods

### 3.1. Data-Driven Remaining Life Prediction Framework

**Figure 1:**
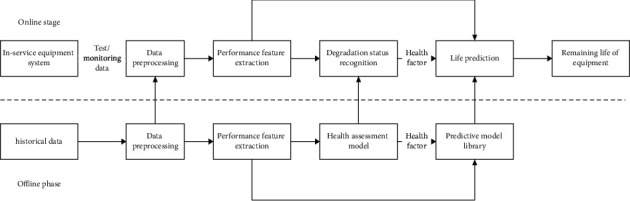
General framework for data-driven remaining life prediction.

A relatively complete data-driven RUL prediction framework suitable for electromechanical sensor equipment systems often covers two stages of online application and offline modeling. The online and offline parts complement and support each other. In the offline phase, make full use of equipment system test data, historical monitoring data, physical and mathematical models of subsystems or components, and so on to establish operating condition recognition, data preprocessing, feature engineering, degradation status recognition, and RUL for online practical applications. In the online stage, after necessary data preprocessing, feature engineering and other operations are performed on the data collected in real-time monitoring, based on the actual operating environment of the service equipment system (such as external environment, load conditions, etc.), with the help of intelligent algorithmic prediction system RUL. In this framework, training and learning in the offline phase and actual prediction in the online phase are fully combined, and the model can be adjusted in an offline or online dynamic manner [16–20], as shown in Figure 1.

Data-driven RUL prediction methods are currently mainly based on machine learning, mathematical statistics, and deep learning. Different prediction methods have different adaptabilities. Corresponding to condition monitoring data, there are usually two RUL prediction strategies. One is to map *m*-dimensional features to a single-dimensional HI value curve and use curve fitting, extrapolation, and other methods to predict, which is called RUL indirect prediction strategy. This article uses direct pattern matching on the original multidimensional data and extracts the *m*-dimensional characterization of degraded fault features to predict the remaining life of the equipment system, which is an RUL direct prediction strategy.

### 3.2. Deep Neural Network

**Figure 2:**
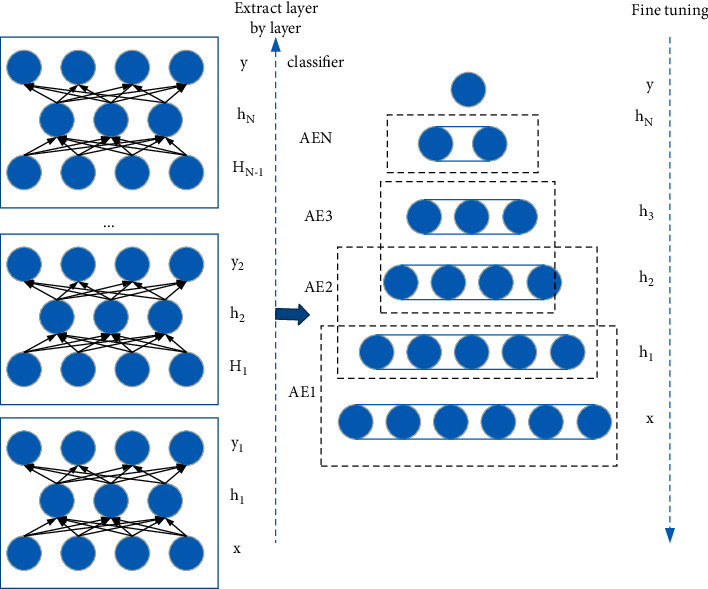
Deep neural network model composed of multiple AEs.

A deep neural network can be regarded as a multihidden neural network formed by stacking multiple AEs. It uses bottom-up unsupervised learning to extract features layer by layer and uses supervised learning methods to fine-tune the parameters of the entire network. The most essential characteristics of a certain state of the object are extracted from the original data, and the deep neural network structure is shown in Figure 2 [21].

#### 3.2.1. Autoencoder

**Figure 3:**
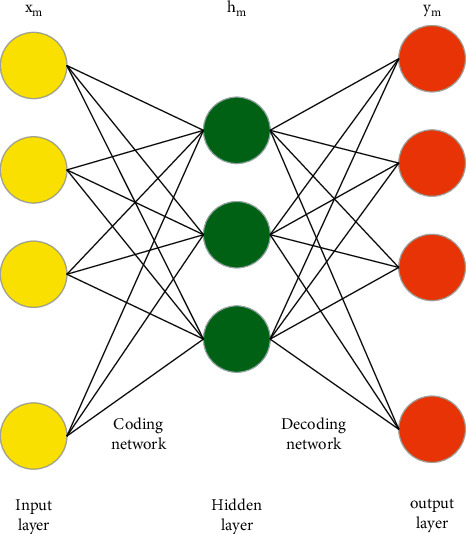
Autoencoder model.

The autoencoder is a three-layer forward artificial neural network, as shown in Figure 3. Its output is equal to the input, and it is mainly composed of an input layer, a hidden layer, and an output layer. The autoencoder is mainly composed of two parts: encoder and decoder. The input data are transformed into features through the encoder network, and the input data are encoded from high- to low-dimensional space data, and the input data are obtained in low-dimensional space. Then, the low-dimensional space data are mapped to the high-dimensional space through the decoder network to realize the reconstruction of the input to the output, and the obtained low-dimensional space data can be used as the feature representation of the input high-dimensional space data [22].

Given an unlabeled data set containing *P* observation variables and *M* samples { x_*pm*_}, (*p* = 1, 2,…, *p*; *m* = 1, 2,…, *M*), the coding network passes The activation function *f*_*θ*_ encodes the sample x_*m*_ into the activation value *h*_*m*_ of the hidden layer, and the coding process is shown in the following equation:

(1) $$\begin{array}{c} h_{m}=f_{\theta }\left(x_{m}\right)=\sigma \left(Wx_{m}+b\right). \end{array}$$

*f*_*θ*_ is the coding function, the function *s* usually takes the Sigmoid function as the activation function of the coding network, *W* is the network weight matrix between the input layer and the hidden layer, *b* is the bias vector generated by the coding network, *θ* = {*W*, *b*} is the connection weight and bias parameter between the input layer and the hidden layer. The general form of the Sigmoid function is shown in the following formula:

(2) $$\begin{array}{c} \sigma x=\frac{1}{1+e^{-x}}. \end{array}$$

Similarly, for the decoding network, the encoding vector *h* obtained by the encoding network is reconstructed by the decoding network to obtain *m*_*y*_ that is equal to the input, that is, *m*_*y*_ and the input *m*_*x*_ are equal. The decoding process is shown in the following equation:

(3) $$\begin{array}{c} y_{m}=g\theta^{T}\left(h_{m}\right)=\sigma \left(W^{T}h_{m}+d\right). \end{array}$$

*g*_*θ* ^*T*^_ is the decoding function, *s* is the activation function of the encoding process, *W*^*T*^ is the network weight matrix from the hidden layer to the output layer, and *d* is the bias vector generated during the encoding process. The essence of the process of training AE is to train and optimize the network parameters *θ* and *θ*^*T*^. In order to make the output *y*_*m*_ as close as possible to the input *x*_*m*_, by minimizing the reconstruction error *J*(*θ*, *θ*^*T*^) (*x*, *y*, *W*, *b*) to characterize the closeness between the input and the output, as shown in the following equation:

(4) $$\begin{array}{c} J_{\left(\theta ,\theta^{T}\right)\left(x,y,W,b\right)}=\frac{1}{m}y-x^{2}. \end{array}$$

In each training process, the gradient descent method is used to update the network training parameters *θ* and *θ*^*T*^ of AE. The entire parameter update process is as follows:

(5) $$\begin{array}{c} W_{1}=W_{1}-\alpha \frac{\partial }{\partial W_{1}}J_{\left(\theta ,\theta^{T}\right)}\left(x,y,W,b\right),\;l=1,2,\\ b_{1}=b_{1}-\alpha \frac{\partial }{\partial W_{1}}J_{\left(\theta ,\theta^{T}\right)}\left(x,y,W,b\right),\;l=1,2. \end{array}$$

*α* is the learning rate and *α*∂/∂*W*_1_*J*_(*θ*, *θ*^*T*^)_(*x*, *y*, *W*, *b*) and *α*∂/∂*W*_1_*J*_(*θ*, *θ*^*T*^)_(*x*, *y*, *W*, *b*) are calculated using backpropagation algorithm.

#### 3.2.2. Deep Neural Network Training

First, the DNN network is pretrained for the failure prediction of electromechanical sensor equipment through the training algorithm. Train the first autoencoder AE_1_ by giving an unlabeled input data set *x* as the input of the coding network and get the coding vector *h*_1_. Set *x* as the output of AE_1_ to obtain the training parameter *θ*_1_; and then use the hidden layer feature *h*_1_ of the first autoencoder as the input of the second autoencoder AE_2_; and train to obtain the network parameter *θ*2 of AE_2_ and *h*_2_ as the hidden layer of AE_2_. The data can be viewed as a feature representation of AE_2_. Repeat this process to obtain the hidden layer feature *h*_*N*_ of the *N*-th autoencoder AE_*N*_ and the corresponding network training parameter *θ*_*N*_ [23, 24].

Second, add a classifier to the top of the DNN network. The pretraining process of the DNN is completed through the layer-by-layer unsupervised training method, which realizes the layer-by-layer extraction of feature information. However, the DNN at this time does not have a classification function. In order to achieve the output classification function, a classifier needs to be added to the top layer of the DNN. This paper uses the softmax classifier as the output layer of DNN. Assuming that the training data set is {x_*m*_}(*m* = 1,2,…, *M*), the label is *u*_*m*_∈{1, 2,…, k}; each probability p(*u* = *i|x*) of a type *i*(*i* = 1,2,…, k) can be calculated by the following hypothesis function:

(6) $$\begin{array}{c} h_{\theta }x_{m}\left[\begin{array}{c} pu_{m}=1|x_{m};\theta \\ pu_{m}=2|x_{m};\theta \\ \vdots \\ pu_{m}=k|x_{m};\theta \end{array}\right]=\frac{1}{\sum_{i=1}^{k}e^{\theta_{i}^{T}}x_{m}}\left[\begin{array}{c} e^{\theta_{1}^{T}}x_{m}\\ e^{\theta_{2}^{T}}x_{m}\\ \vdots \\ e^{\theta_{k}^{T}}x_{m} \end{array}\right]. \end{array}$$

*θ* is the model parameter of softmax, which is similar to the AE model. In order to ensure the performance of the classifier, the training parameters of the model are optimized by minimizing the loss function *J*_*θ*_. The top-level network parameter *θN* + 1 can be obtained by minimizing *J*_*θ*_*x*_*m*_ as follows:

(7) $$\begin{array}{c} J_{\theta }x_{m}=-\frac{1}{M}\left[\sum_{m=1}^{M}\sum_{m=1}^{M}1\left\{u_{m}=i\right\}\log\frac{e^{\theta_{i}^{T}}x_{m}}{\sum_{i=1}^{k}e^{\theta_{i}^{T}}x_{m}}\right]. \end{array}$$

Finally, fine-tune the training parameters of the entire DNN. In order to ensure the accuracy of feature extraction and the classification effect of the output layer, the backpropagation algorithm is used to supervise the fine-tuning of the entire DNN training parameters through a limited number of sample labels, and the fine-tuning process is completed by minimizing the reconstruction error *E*(*θ*). The parameter update process is as follows:

(8) $$\begin{array}{c} E\left(\theta \right)=\frac{1}{M}\sum J_{\theta }\left(Y_{m},u_{m};\theta \right),\\ \theta =\theta -\alpha \frac{\partial E\left(\theta \right)}{\partial \left(\theta \right)}. \end{array}$$

*Y*_*m*_ is the true output value, *θ* is the parameter set obtained from the entire network training process *θ* = {*θ*_1_, *θ*_*2*_,…, *θ*_*N*_, *θ*_*N+1*_}, the back-propagation algorithm is mainly used to continuously modify the network parameters  *θ*, and *α* is the learning rate. The fine-tuning process uses labeled data to improve the accuracy of DNN training.

## 4. Based on the Improved Residual Neural Network Electromechanical Sensor Equipment Fault Diagnosis Model System Design

### 4.1. Random Weight Average Method SWA

#### 4.1.1. Learning Rate

During SGD training, there are two strategies for setting the learning rate: fixed learning rate and periodic learning rate. Under a certain learning rate strategy of SWA, the average of the weight data in the SDP training process is used as the weight algorithm. When the environment of the learning rate changes, assuming that the learning rate decreases linearly from *α*_1_ to *α*_2_ in a cycle, the change formula of the learning rate is

(9) $$\begin{array}{c} \alpha \left(i\right)=1-ti\alpha_{1}+t\left(i\right)\alpha_{2}ti=\frac{1}{c}\left(\text{mod}\left(i-1,c\right)+1\right). \end{array}$$

#### 4.1.2. Learning Rate Error Curve

**Figure 4:**
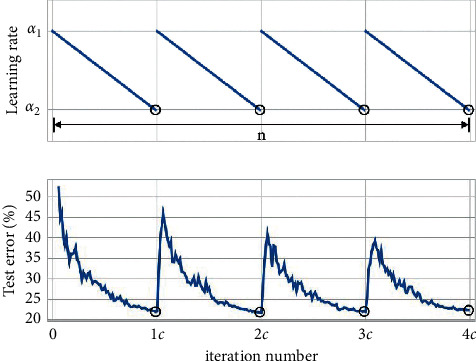
Periodic change learning rate and its training error rate.

The performance of the learning rate and error rate during the training of the SGD algorithm is shown in Figure 4.

In Figures 1–4, the picture shows the change curve of the learning rate, and the picture below shows the training error corresponding to the learning rate. The small circle on the learning rate curve indicates the minimum learning rate and its corresponding test error. Figure 4 shows the change in the learning rate and the corresponding error rate during a batch of training. It can be found that the learning rate does not change continuously. At the end of a cycle, the learning rate jumps directly from the minimum to the maximum. Basically, the learning rate *α*_1_ ≥ *α*_2_, and the length *c* of a cycle is a hyperparameter. In order to improve training, it is also necessary to consider the case where the learning rate remains unchanged.

### 4.2. Dropout Regularization

**Figure 5:**
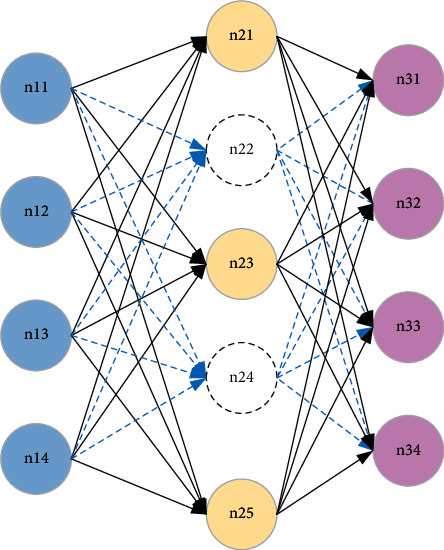
Network structure using dropout.

If the training sample is insufficient, it is easy to produce overfitting phenomenon. The phenomenon of overfitting will reduce the effect of actual prediction. The failure prediction model of electromechanical sensor equipment has a smaller loss on the training set and a higher accuracy rate, while the opposite is true on the test machine. Dropout not only can effectively reduce the overfitting phenomenon but also can reduce the fault prediction error rate of electromechanical sensor equipment. Its essence is that during training, the neuron becomes 0 with probability *p* and does not participate in the process of forward propagation. This brings about a reduction in the dependence of the electromechanical sensor equipment failure prediction model on local features, so the electromechanical sensor equipment failure prediction model has better generalization capabilities. The structure of dropout is shown in Figure 5.

#### 4.2.1. Workflow of Dropout

1. Set the node hidden probability 0 <*p* < 1, and the neurons in the hidden layer will be shielded with probability *p*
2. The neurons in the hidden layer are shielded with probability *p*, and the shielded neurons no longer participate in the training process
3. Neurons that have not been deleted proceed forward and backward in the normal way and update normally (w, *b*)
4. Restore the shielded neurons and then perform steps 1 and 2

From the above analysis, we can know that the idea of dropout is that certain neurons do not participate in training at certain times.

#### 4.2.2. How to Implement Dropout

**Figure 6:**
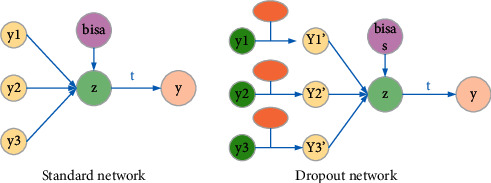
Comparison of the ordinary network and dropout layer.

During the training phase, each neuron needs to be shut down probabilistically. The model diagram is shown in Figure 6. The corresponding calculation process is as follows.

When there is no dropout layer, the calculation becomes

(10) $$\begin{array}{c} z_{i}^{\left(l+1\right)}=w_{i}^{\left(l+1\right)}y^{l}+b_{i}^{\left(l+1\right)}. \end{array}$$

The corresponding network calculation formula without dropout is as follows:

(11) $$\begin{array}{c} r_{i}^{\left(l\right)}∼\text{Bernoulli}\left(p\right), \end{array}$$

(12) $$\begin{array}{c} y^{丿\left(l\right)}=r^{\left(l\right)}\times y^{\left(l\right)}, \end{array}$$

(13) $$\begin{array}{c} z_{i}^{\left(l+1\right)}=w_{i}^{\left(l+1\right)}y^{丿\left(l\right)}+b_{i}^{\left(l+1\right)}, \end{array}$$

(14) $$\begin{array}{c} y_{i}^{\left(l+1\right)}=f\left(z_{i}^{\left(l+1\right)}\right). \end{array}$$

Equations (12)–(14) use Bernoulli functions. The function of the Bernoulli function is to generate a random vector composed of 0 and 1 according to the probability. In addition, *f* represents the activation function; *y* represents the output; *z* represents the value of the neuron after the summation, which becomes the output after the activation function.

#### 4.2.3. Effectiveness Analysis of Dropout

**Figure 7:**
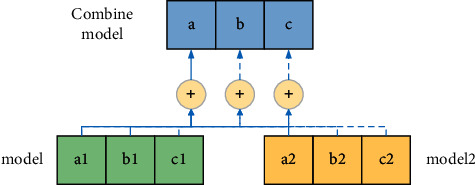
The fusion of the two models.

For model optimization, there is a classic method. For a specific electromechanical sensor device to predict the scene, train multiple models at the same time, as shown in Figure 7. This is a three-category model that predicts three categories of *a*/*b*/*c*, among which

(15) $$\begin{array}{c} \alpha =\frac{\alpha_{1+}\alpha_{2}}{2},\\ b=\frac{b_{1+}b_{2}}{2}, \end{array}$$

(16) $$\begin{array}{c} c=\frac{c_{1+}c_{2}}{2}. \end{array}$$

Figure 7 shows a structure in which two models are averaged. Through the superposition of multiple networks, the generalization ability of the predictive model of electromechanical sensor equipment can be improved. And dropout achieves the effect of multiple networks superimposed to a certain extent. Because after regularization, the network will randomly lose some neurons each time, the model structure after discarding is different from that before discarding, which is equivalent to a new network. In this way, there will be many different networks, and then the final prediction results will be obtained by averaging or voting on these models. Dropout replaces the multimodel solution to a certain extent. From the above analysis, we know that some neurons will be randomly closed every time forward and backward. In essence, the neurons can be considered as a new network after they are closed. In this way, multiple networks are generated during the training process, and the final prediction result is the average effect of multiple networks. After taking the average, the risk of overfitting of the model is effectively suppressed. With the introduction of dropout, two neurons may not appear in the network at the same time. In this way, the weight update does not depend on the joint action of all hidden nodes. In this way, the problem of excessive reliance on special features in the training process is prevented. Make the neural network train some more common features for classification. Increase the robustness of the network. It can also be said that the introduction of dropout reduces the sensitivity of special features during the training process so that the generalization ability of the system is significantly improved. The dropout layer prevents some nodes from participating in training so that the actual training time of the node is less than the set algebra, which also reduces the risk of overfitting in the prediction process of electromechanical sensor equipment.

## 5. Fault Prediction and Analysis of Electromechanical Sensors Based on Improved Residual Neural Network

### 5.1. Operation of Electromechanical Sensor Fault Prediction System Operation

**Figure 8:**
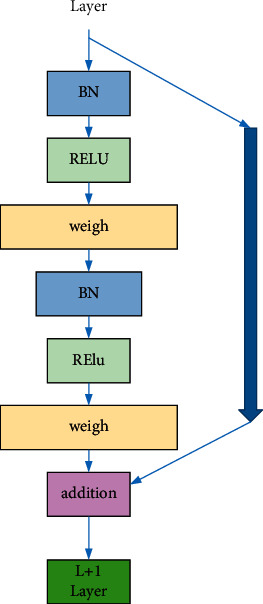
Structure realization of the residual block.

For the residual neural network, building the residual block is the most fundamental task of building a residual neural network. A good residual block design is very important for neural networks. A typical residual block contains two convolutional layers, and the two convolutional layers are not directly connected. In order to improve the recognition effect of the algorithm, we introduced the BN layer and the activation function, and the combination between them constitutes a complete residual layer. There are multiple combinations of residual blocks, and the residual structure selected in this paper is shown in Figure 8 [25, 26].

**Figure 9:**
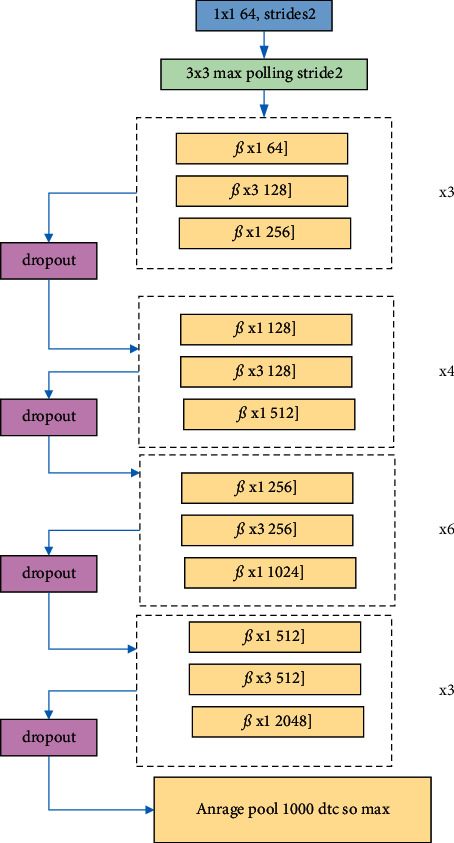
ResNet50 network structure after adding dropout.

This paper analyzes the predictions of electromechanical sensor equipment for improved networks, optimized by swa algorithm, dropout regularization, and both algorithms. After the dropout optimization of the ResNet50 network structure, its network structure becomes Figure 9. In ResNet50, a total of five dropout structures are added.

### 5.2. Result Analysis

**Figure 10:**
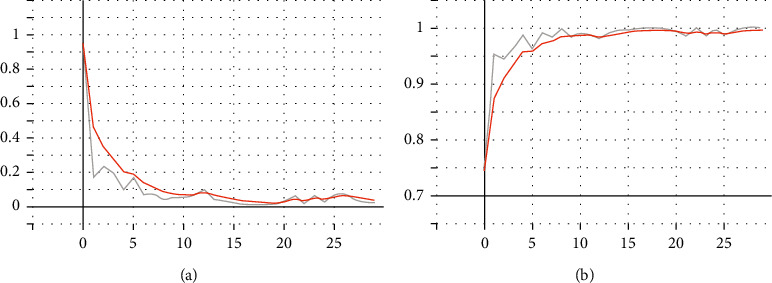
ResNet training effect, left loss, and right Acc before the improvement.

This paper randomly selects 30 fault features; the training algebra is set to 30 generations; and the trained electromechanical sensor equipment prediction model is ResNet50 and its improved model. After comparison of experiments, it is found that the four kinds of electromechanical sensor equipment prediction models trained have overfitting, but the difference is that their overfitting degrees are inconsistent. The following are the experimental results: the training results of the original ResNet50 are shown in Figure 10.

The training results are as follows: The training accuracy rate is 99.84%, and the loss is 0.03. The training accuracy rate is 91.69%, and the loss is 0.49.

**Figure 11:**
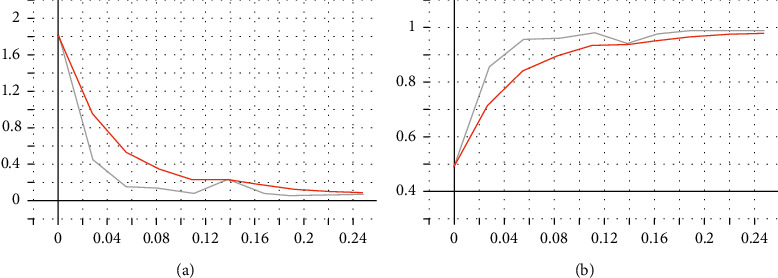
ResNet training effect, left loss, and right Acc after adding dropout regularization.

After adding dropout regularization, the training effect is shown in Figure 11.

The training results are as follows: The training accuracy rate is 99.93%, and the loss is 0.04. The training accuracy rate is 92.75%, and the loss is 0.67.

**Figure 12:**
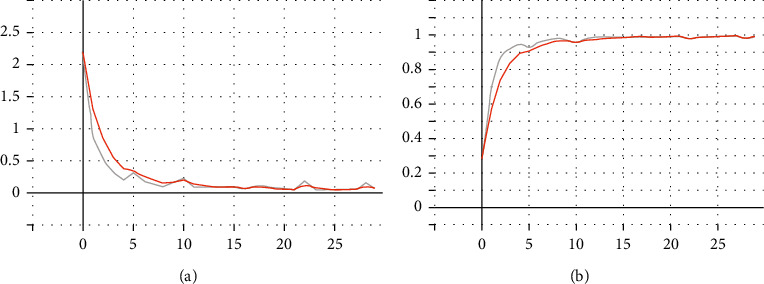
swa improved ResNet50 training results.

After improving the model through the swa algorithm, the training effect diagram is shown in Figure 12.

The training results are as follows: The training accuracy rate is 99.82%; loss is 0.02.

**Figure 13:**
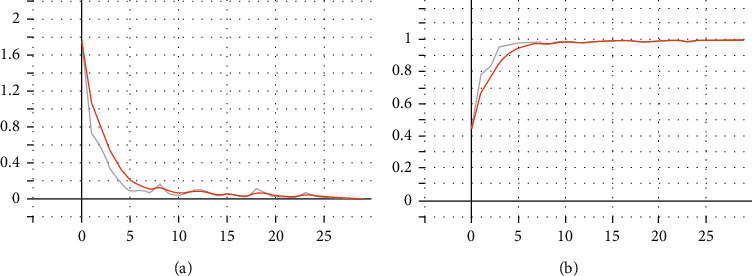
Improved swa and dropout, ResNet50 training results.

The training accuracy rate is 95.19%; loss is 0.54. After adding dropout regularization and swa algorithm improvement, the training effect diagram is shown in Figure 13.

The training results are as follows: The training accuracy rate is 99.51%, and the loss is 0.3. The training accuracy rate is 95.61%, and the loss is 0.44.

Through the above data verification, the following conclusions can be drawn:

1. The four electromechanical sensor equipment prediction models all have an overfitting phenomenon, that is, the test effect of the test set is significantly lower than the training effect of the training set.
2. To the network that joins the dropout, the training process fluctuates greatly, which is in line with the expected result. Because when the network turns off certain weights, it is equivalent to a new network, and training on this basis may fluctuate.
3. Networks that use swa algorithm improvement and simultaneous swa algorithm and dropout regularization algorithm improvement have the best test results, but the latter is slightly better. This shows that the random weight averaging algorithm and dropout have the effect of suppressing overfitting.

## 6. Conclusion

This article takes electromechanical sensor equipment as the research object. Aiming at the situation of strong human intervention and insufficient recognition ability in the fault diagnosis of electromechanical sensor equipment, the study of deep learning in the fault diagnosis of electromechanical sensor equipment is carried out. The work of this article is summarized as follows:

The advanced convolutional neural network model is used to maximize the use of the fault recognition ability of the deep learning network for fault diagnosis, reducing manual intervention models.

Aiming at the phenomenon that the electromechanical sensor equipment predicts that the training process is prone to overfitting, two improved methods are adopted. Based on the random weight averaging algorithm and dropout regularization, the model is improved from two aspects: backpropagation and model structure. Experiments show that when the backpropagation learning rate is fixed or periodically changes, the SWA algorithm can effectively improve the training effect. Dropout regularization is to randomly discard some neurons so that the network will not rely too much on certain local features, thereby improving the generalization ability of the model to suppress overfitting, and the effect of electromechanical sensor equipment failure prediction is better.

## Data Availability

The data set can be accessed upon request to the corresponding author.
